# Mitochondrial pathways and sarcopenia in the geroscience era

**DOI:** 10.1016/j.jnha.2024.100397

**Published:** 2024-10-19

**Authors:** Emanuele Marzetti, Riccardo Calvani, Helio José Coelho-Junior, Anna Picca

**Affiliations:** a Department of Geriatrics, Orthopedics and Rheumatology, Università Cattolica del Sacro Cuore, Rome, Italy; b Fondazione Policlinico Universitario “Agostino Gemelli” IRCCS, Rome, Italy; c Department of Medicine and Surgery, LUM University, Casamassima, Italy

**Keywords:** Biology of aging, Extracellular vesicles, Inflammaging, Mitochondrial quality control, Multi-Marker, Omics

## Abstract

Sarcopenia is associated with structural, ultrastructural, and molecular abnormalities of skeletal muscle. Mitochondrial dysfunction is a pivotal factor involved in muscle aging and sarcopenia. Mitochondrial bioenergetics are significantly reduced in muscles of older adults which is associated with whole-body aerobic capacity, muscle strength, and physical performance. Transcriptional profiling of muscle samples from older adults also revealed inverse correlations between gene expression patterns of autophagy and mitophagy and muscle volume and physical performance. This is in line with the proposition that mitochondrial quality control (MQC) processes are key to organellar and tissue health. MQC encompasses mitochondrial biogenesis, dynamics, and mitophagy. The latter has recently been included among the hallmarks of aging and alterations in MQC have been associated with chronic sterile inflammation as well as muscle atrophy and dysfunction. Several biomarkers spanning MQC, inflammation, metabolism, intercellular communication, and gut microbiota have been linked to sarcopenia. Findings from these initial studies hold promise to inform geroscience-based research in the field of sarcopenia by offering a plausible biological framework for developing gerotherapeutics and monitoring their effects.

## 1 Introduction

Sarcopenia is characterized by a progressive, age-related loss of muscle mass, strength, and function [1]. These changes are often accompanied and partly mediated by structural alterations within the muscle, including the conversion of type 2 (fast-twitch glycolytic fibers that produce high force for short durations) to type 1 fibers (slow-twitch oxidative fibers that produce low force for extended periods), as well as inter- and intramuscular fat infiltration [1].

Numerous intrinsic and extrinsic mechanisms have been associated with sarcopenia, but mitochondrial function and associated signaling pathways have emerged as critical factors [2]. Mitochondria are a major source of reactive oxygen species (ROS), which are generated as a byproduct of oxidative phosphorylation [3]. The mitochondrial theory of aging posits that ROS-induced damage accumulates over a lifetime, affecting lipids, proteins, and nucleic acids, ultimately disrupting mitochondrial integrity and creating a vicious cycle where dysfunctional mitochondria produce more ROS [3]. Although this theory has been revised, it forms the basis of the view that aging results from the continuous balance between damage accumulation and resilience potential [4]. As resilience diminishes with age, individuals become more prone to a phenotype characterized as frailty [4].

The relevance of mitochondrial dysfunction to muscle aging is supported by evidence of significantly reduced mitochondrial respiration in the muscles of older adults [5]. Notably, mitochondrial respiratory capacity correlates with whole-body aerobic capacity, muscle strength, and physical performance [5]. Subsequent studies have reinforced the association between muscle mitochondrial bioenergetics and muscle power, physical performance, and fatigability in older adults [6,7]. Transcriptional profiling has also revealed that autophagy and mitophagy gene expression patterns in muscle are inversely correlated with physical performance, muscle volume, and mitochondrial function [8]. These findings align with the notion that mitochondrial quality control (MQC) processes—encompassing mitochondrial biogenesis, dynamics, and mitophagy—are essential for maintaining organellar and tissue health [9]. Disruptions in MQC have been linked to muscle atrophy and dysfunction in both preclinical models and humans [10]. For instance, altered expression of markers for mitochondrial biogenesis, dynamics, and the autophagy/mitophagy-lysosomal system has been associated with muscle dysfunction, sarcopenia, and deterioration of lower extremity tissue composition in older adults [10]. These observations indicate that mitochondria and MQC processes are promising biological targets for interventions against sarcopenia.

## 2 Exercise: multiple magic bullets against sarcopenia

Exercise is one of the most effective interventions to foster healthy aging [11]. Its impact on aging muscle is multifaceted, involving the promotion of satellite cell proliferation, myogenesis, nitric oxide-guided vascularization, improvements in mitochondrial bioenergetics, and reduced myonuclear apoptosis [7,11]. A recent investigation from the Study of Muscle, Mobility and Aging (SOMMA) has demonstrated a strong association between skeletal muscle bioenergetics and physical activity [7]. Each additional 30 min per day of accelerometer-measured moderate-to-vigorous physical activity (MVPA) was associated with higher maximal mitochondrial oxidative phosphorylation (maxOXPHOS) and greater ATP maximal production (ATPmax) [7]. Conversely, each 30-minute period of sedentary behavior was associated with decreased maxOXPHOS and ATPmax [7]. Moreover, MVPA attenuated the age-dependent decline in ATPmax, particularly in men [7]. Collectively, these findings support the link between mitochondrial dysfunction and muscle aging, emphasizing the role of regular exercise in enhancing mitochondrial capacity [12].

## 3 A geroscience approach to the study of muscle aging: Facts and perspectives

The geroscience hypothesis proposes that advancing age is the primary risk factor for most non-communicable diseases [13]. This paradigm has ignited research efforts to identify reliable and accessible biomarkers of aging that can evaluate the effects of geroscience-based interventions. In older adults with sarcopenia, several circulating biomolecules involved in inflammation, muscle growth and remodeling, neuromuscular junction damage, and amino acid metabolism have been identified and proposed as potential biomarkers [14]. Additionally, specific microbial signatures have been discovered in fecal samples of older individuals with sarcopenia [15], while distinct metabolomic profiles have been observed in older adults with physical frailty and sarcopenia compared to pre-frail or frail peers with type 2 diabetes [16].

Further investigation into the molecular mechanisms of aging is essential for identifying novel biomarkers of muscle aging. For example, recent evidence suggests that “inflammaging” may result partly from the extracellular release of molecules that activate damage-associated molecular patterns [17]. Among these are products of piecemeal mitophagy, which are released by cells within mitochondria-derived vesicles [17]. Quantifying circulating extracellular vesicles and characterizing their cargo have allowed specific signatures to be identified in older adults with physical frailty and sarcopenia [18]. Moreover, distinct profiles of molecules related to the senescence-associated secretory phenotype (SASP)—including markers of inflammation, extracellular matrix remodeling, and mitochondrial dysfunction—have been identified as molecular signatures of physical frailty and sarcopenia [19].

Although preliminary, these findings provide a biological framework for geroscience-based research in sarcopenia and present opportunities for developing gerotherapeutics and monitoring their effects [13,20].

## 4 Conclusion and perspectives

Age-related declines in mitochondrial function and disruptions in MQC are implicated in muscle aging and the pathogenesis of sarcopenia and physical frailty. Interventions incorporating MVPA are critical in mitigating the rate and magnitude of age-related declines in muscle mitochondrial bioenergetics. Empirical evidence indicates that physical activity and exercise effectively prevent adverse outcomes associated with sarcopenia and physical frailty. The simultaneous analysis of multiple biomolecules enables a deeper understanding of muscle aging through the identification of biologically plausible biomarker patterns across various physiological domains. Further elucidation of the molecular pathways underlying the aging process will facilitate the identification of novel drug targets to treat sarcopenia and the development of new biomarkers to monitor geroscience-based interventions.

## Declaration of Generative AI and AI-assisted technologies in the writing process

No Generative AI or AI-assisted technologies were used in the writing process.

## Funding

This work was supported by Innovative Medicine Initiative-Joint Undertaking (IMI-JU #115621).

## Declaration of competing interest

The authors declare that they have no known competing financial interests or personal relationships that could have appeared to influence the work reported in this paper.
